# Propriospinal Neurons: Essential Elements of Locomotor Control in the Intact and Possibly the Injured Spinal Cord

**DOI:** 10.3389/fncel.2019.00512

**Published:** 2019-11-12

**Authors:** Alex M. Laliberte, Sara Goltash, Nicolas R. Lalonde, Tuan Vu Bui

**Affiliations:** Department of Biology, Faculty of Science, Brain and Mind Research Institute, University of Ottawa, Ottawa, ON, Canada

**Keywords:** propriospinal neurons, spinal locomotor networks, central pattern generators, spinal cord injury, detour circuits

## Abstract

Propriospinal interneurons (INs) communicate information over short and long distances within the spinal cord. They act to coordinate different parts of the body by linking motor circuits that control muscles across the forelimbs, trunk, and hindlimbs. Their role in coordinating locomotor circuits near and far may be invaluable to the recovery of locomotor function lost due to injury to the spinal cord where the flow of motor commands from the brain and brainstem to spinal motor circuits is disrupted. The formation and activation of circuits established by spared propriospinal INs may promote the re-emergence of locomotion. In light of progress made in animal models of spinal cord injury (SCI) and in human patients, we discuss the role of propriospinal INs in the intact spinal cord and describe recent studies investigating the assembly and/or activation of propriospinal circuits to promote recovery of locomotion following SCI.

## Introduction

Successful locomotion depends upon the precise coordination of multiple muscles across numerous joints and limbs, as well as the simultaneous engagement of multiple trunk and stabilizing muscles (Frigon, 2017). This patterned motor output must be adjusted dynamically at differing speeds and in response to various obstacles and perturbations, requiring the constant integration of sensory information. While the role of supraspinal centers, particularly in relation to the planning, initiation, and modulation of locomotion (Lajoie and Drew, 2007; Capelli et al., 2017; Caggiano et al., 2018; Josset et al., 2018; Oueghlani et al., 2018), should not be discounted, many of the key functions of locomotion are performed by interneuron (IN) networks within the spinal cord. This is particularly evident from experiments utilizing *ex vivo* spinal cord preparations where application of electrical stimulation, light illumination, or a variety of neurotransmitter/pharmacological agonist cocktails have successfully evoked fictive locomotion – sustained rhythmic and appropriately patterned flexor and extensor activity recorded in motor nerves of the spinal cord – in the absence of supraspinal input (Atsuta et al., 1991; Jiang et al., 1999; Whelan et al., 2000; Hägglund et al., 2010). The innate potential of select propriospinal INs, defined as spinal cord INs that originate and terminate within the spinal cord while spanning at least one spinal cord segment, to activate locomotion makes them an attractive therapeutic target for spinal cord injury (SCI) (Flynn et al., 2011). However, an incomplete understanding of their integration within locomotor systems remains a significant obstacle to the use of propriospinal INs in the therapies seeking to restore lost locomotor function. Emergent genetic and molecular techniques that allow the identification and manipulation of specific propriospinal IN populations have greatly accelerated this research. It is the purpose of this review to summarize and update the current state of knowledge regarding the organization and function of locomotion-associated propriospinal INs in intact and spinal cord lesioned mammals as well as to examine seminal and recent attempts to manipulate propriospinal INs to rescue locomotor function.

## Propriospinal Interneurons Propagate Locomotor Commands From Supraspinal Locomotor Regions

**FIGURE 1:**
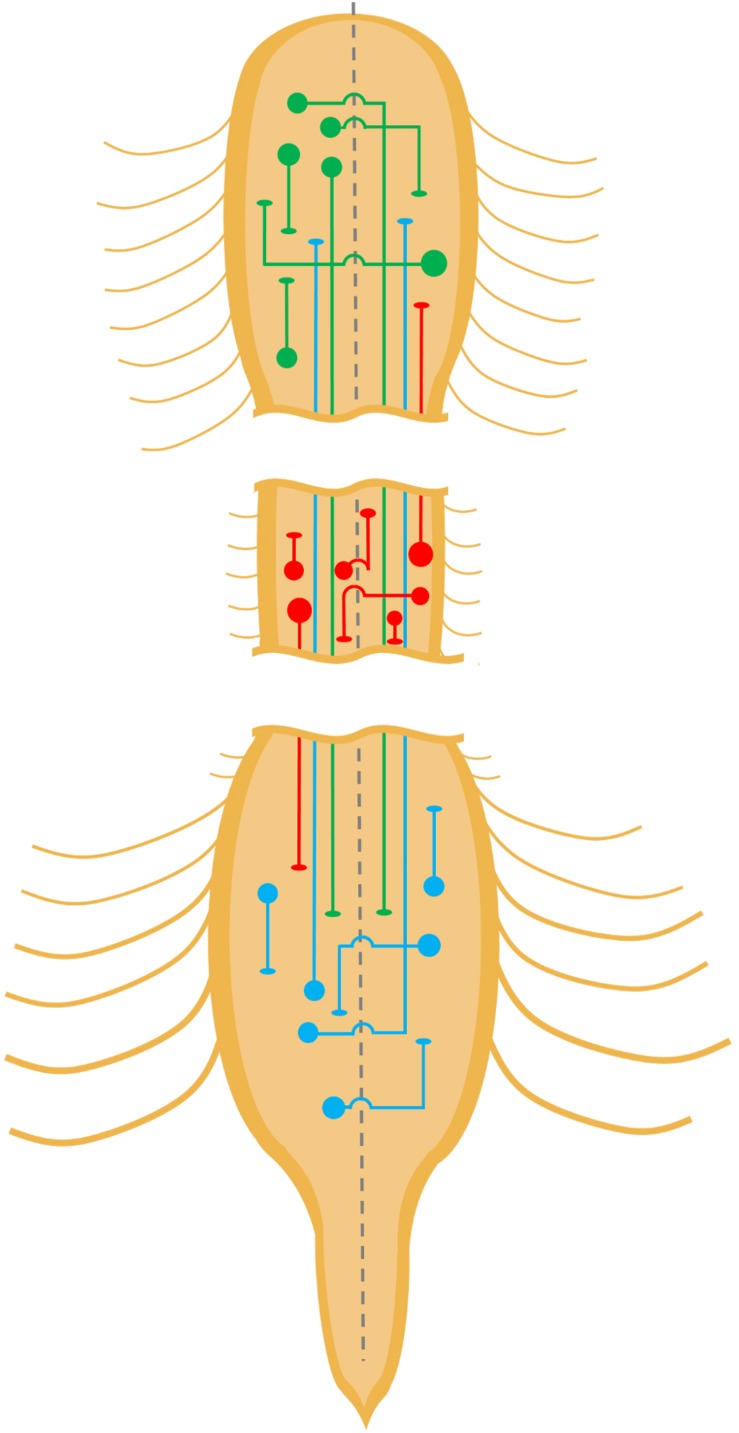
Propriospinal neurons of the spinal cord. Schematic diagram depicting several types of propriospinal neurons within the cervical, thoracic, and lumbar spinal cords. For the sake of simplicity, some types are not depicted such as bifurcating commissural interneurons with axon collaterals that ascend and descend. Depicted medio-lateral locations of different types of priopriospinal neurons or their terminations are not meant to be accurate representations of known localizations.

**FIGURE 2:**
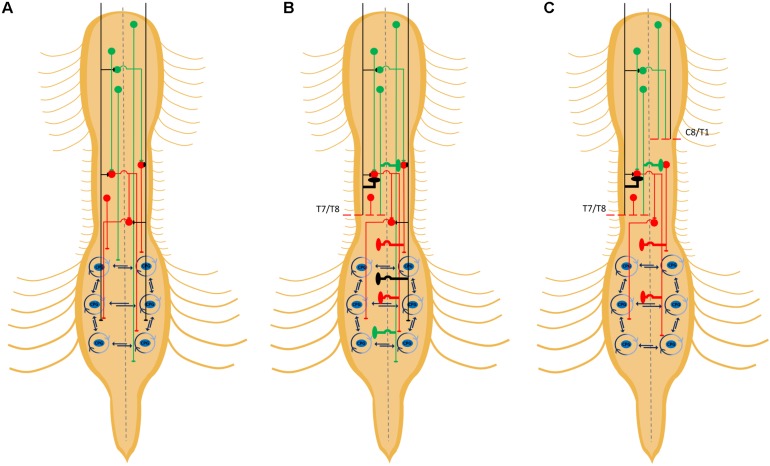
Detour circuits arising from staggered hemisections. Schematic diagram depicting some propriospinal circuits innervating lumbar central pattern generators (CPGs) **(A)**, some possible detour circuits formed after an initial hemisection **(B)**, and spared detour circuits following a second, delayed, contralateral hemisection **(C)**. Collateral sprouting depicted by bold lines. The propriospinal neurons within the lumbar spinal cord are not depicted but would either be connected to, or part of, CPGs. Descending inputs from detour circuits projecting to the lumbar spinal cord can project to lumbar propriospinal neurons and/or lumbar CPGs.

At first glance, propriospinal INs occupy a conceptually straightforward role in locomotion. Propriospinal INs receive inputs from descending locomotor pathways and propagate received motor commands rostrocaudally to locomotor circuits via short or long, ipsilateral or commissural axons (Figure 2A). To fulfill this role, some propriospinal INs project axons that only travel a few segments (short propriospinal), while others project axons that travel many more segments, spanning far enough to connect cervical with lumbar segments (long propriospinal). Short and long propriospinal IN axons can stay within the same side of the body as their cell bodies (Figure 1, straight lines), while others cross to the other side (Figure 1, lines with arc segments). We include commissural INs for their possible involvement in forming detour circuits following an injury to the spinal cord. Evidence for the role of propriospinal INs in propagating locomotor commands rostrocaudally has been well-established in the mammalian CNS. For example, the application of certain neurotransmitter agonists to only the cervicothoracic spinal cord was found to generate rhythmic hindlimb activity in neonatal rat preparations (Cowley and Schmidt, 1997). Transections at caudal thoracic and rostral lumbar segments also abolished locomotor activity below the caudal lesion site, demonstrating the importance of INs in this region for locomotor output in more caudal lumbar segments (Cowley and Schmidt, 1997). Additional evidence for propriospinal locomotor relays was demonstrated using brainstem stimulation in rat brainstem-spinal cord *in vitro* preparations, where hindlimb locomotor-like activity was observed despite staggered contralateral spinal cord lesions that severed all ipsilateral bulbospinal connections on both sides of the spinal cord (Cowley et al., 2008). These findings suggest that either brainstem locomotor regions have projections to the contralateral spinal cord that decussate at the spinal levels between the hemisections, or that propriospinal INs with contralateral projections propagate locomotor commands from supraspinal centers (e.g., red lines with arc segments in Figure 1). The latter explanation that propriospinal networks are integrated into bulbospinal locomotor control appears more likely given (1) the predominance of ipsilaterally projecting axons over contralaterally projecting from brainstem locomotor centers (Stelzner and Cullen, 1991; Liang et al., 2011; Sivertsen et al., 2016) and (2) subsequent physiological experiments which found that blockade of synaptic transmission in cervicothoracic segments (using a variety of strategies: no Ca^2+^, high Mg^2+^, CNQX, AP-5) resulted in the abolition of brainstem stimulation-induced lumbar locomotor activity (Zaporozhets et al., 2006).

These studies strongly suggest a role for propriospinal INs in the propagation of locomotor commands, implying that propriospinal INs either innervate or form part of the locomotor central pattern generator (CPG). Indeed, it has been proposed that the locomotor CPG may be composed of multiple unit CPGs distributed along the length of the spinal cord and across its midline controlling subsets of muscles, in particular, those acting upon particular joints such as the hip, knee, or ankle (Grillner et al., 1976; Kjaerulff and Kiehn, 1996; Wiggin et al., 2012; Gerasimenko et al., 2016; Mantziaris et al., 2017). Short propriospinal neurons (Figure 1, short lines, green and blue, straight or with arc segment) may act as relays between non-overlapping unit CPGs within cervical or lumbar segments. Alternatively, short propriospinal INs may be part of unit CPGs if the circuitry for each unit CPG is spread out over several spinal cord segments. In either case, propriospinal connections can propagate locomotor commands from their initial targets to adjacent nodes containing CPG components.

## Propriospinal Interneurons Perform Functions of the Locomotor CPG

Ontogenic studies of molecularly defined populations of propriospinal INs have provided ample evidence of the contributions of propriospinal INs to locomotor CPG function. While others have extensively reviewed the developmental origin of INs comprising the locomotor CPG (Gosgnach et al., 2017), this information is critical to understanding the role of propriospinal INs in locomotor function and therefore, a brief overview of this topic is warranted. It should be noted that each of the developmentally defined IN populations are believed to be heterogeneous to varying degrees, potentially containing several distinct subpopulations of INs with different characteristics. As a result, not all of these subpopulations will meet the currently used definition of propriospinal INs. However, at least some portion of the IN populations discussed below appear to be propriospinal based on their reported projection characteristics (reviewed in Lu et al., 2015), and their putative function in locomotion (Table 1).

**TABLE 1:** Overview of major developmentally defined interneuron populations and their proposed role in locomotion.

**Interneuron population**	**Projection characteristics**	**Neurotransmitter phenotype**	**Putative function in mammalian locomotion**
V0_V_ (*DBX1*+/*EVX1*+)	Long and short commissural	Glutamatergic (excitatory)	Coordinates left–right alternation during locomotion. Loss of function particularly affects alternation during higher speed locomotion.
V0_D_ (*DBX1*+*/PAX7*+)	Long and short commissural	GABA/glycinergic (inhibitory)	Coordinates left–right alternation during locomotion. Loss of function particularly affects alternation during lower speed locomotion.
V0_C_ (*DBX1*+/*EVX1*+*/PITX2*+)	Short ipsilateral and commissural	Cholinergic (excitatory)	Modulates activity of some motoneuron pools during specific locomotor tasks.
V1 (*EN1*+)	Short and long ipsilateral	GABA/glycinergic (inhibitory)	Coordinate flexor–extensor activity, potentially through the inhibition flexor activity. Include Renshaw and Ia IN populations with well-defined roles in recurrent and reciprocal inhibition of motoneurons.
V2a (*CHX10*+)	Long and short ipsilateral	Glutamatergic (excitatory)	Propagate locomotor commands to commissural interneurons involved with left–right coordination.
V2b (*GATA2/3*+)	Long and short ipsilateral	GABA/glycinergic (inhibitory)	Coordinate flexor–extensor activity, potentially through the inhibition of extensor activity.
V3 (*SIM1*+)	Short commissural and ipsilateral	Glutamatergic (excitatory)	Stabilize locomotor pattern, reducing variability in ipsilateral and contralateral gait.
dI3 (*ISL1*+)	Short ipsilateral	Glutamatergic (excitatory)	Relay cutaneous and proprioceptive information to CPG. Essential for locomotor rehabilitation.
dI4 dIL_A_ (*Ptf1a*+)	Short ipsilateral Short commissural	GABAergic (inhibitory)	Mediate presynaptic inhibition of sensory terminals onto spinal neurons to gate sensory feedback and ensure smooth execution of movements.
dI6 (*DMRT3*+ and/or *WT1*+)	Short commissural and ipsilateral	GABA/glycinergic (inhibitory)	Stabilize locomotor pattern, reducing variability in ipsilateral and contralateral gait.

During embryogenesis, the spatiotemporal distribution of signaling molecules such as Wnt, BMP, and Sonic hedgehog, across three spatial axes – rostral–caudal, medial–lateral, and dorsal–ventral – leads to a complex spatiotemporal pattern of transcription factor activation that results in the emergence of a specific set of progenitor pools within the spinal cord (Dasen and Jessell, 2009). In total, there are 13 progenitor pools from which INs and motoneurons will emerge. All dorsal IN populations (dI1–dI6) come from progenitor pools pd1–6 and the late-born pdIL_*A*_ and pdIL_*B*_. Four ventral IN populations (V0–V3) emerge from the p0–3 pool while the motoneurons arise from the motor domain pMN (reviewed in Lu et al., 2015). The ventrally derived populations have received considerable interest in relation to locomotion, as it has been postulated that they constitute the core elements of the CPG located primarily within the spinal ventral horn (reviewed in Kiehn, 2016). Therefore, many genetic manipulations have targeted these ventral IN populations. Initial experiments with isolated spinal cords observed the conservation of rhythmicity following a sagittal section along the midline of chronically isolated adult cat spinal cords (Kato, 1990). This observation seems to indicate that rhythmogenesis can be generated solely from ipsilaterally projecting neurons within each side of the spinal cord. Therefore, the first ablation studies targeted ipsilaterally projecting INs.

The V1 embryonic population of INs, which are marked by the expression of the engrailed-1 (En1) transcription factor, generates inhibitory neurons that project ipsilaterally (Higashijima et al., 2004; Benito-Gonzalez and Alvarez, 2012). Approximately 30% of En1+ INs become Ia INs or Renshaw cells and form inhibitory synapses onto motoneurons or other Ia INs (Alvarez et al., 2005). As the V1/Ia IN population projects ipsilaterally within the spinal cord, targeted ablation of En1+ INs was expected to result in the loss of flexor–extensor intralimb coordination. Instead, loss of V1 neurons resulted in a loss of high-speed locomotor activity. Indeed, mice lacking V1 INs had lower top speeds on the rotarod test compared to their control mates, and the duration of the step cycle was increased in recordings made from isolated spinal cords (Gosgnach et al., 2006). Subsequent experiments using genetic methods to block synaptic transmission from V1 INs instead of developmental ablation recapitulated this finding (Zhang et al., 2014). While V1 IN silencing alone did not prevent flexor–extensor coordination, V1 IN ablation resulted in a prolonged flexion phase in both neonatal and adult mice, while optogenetic activation of V1s resulted in the suppression of flexor activity (Britz et al., 2015). A recent study provided further confirmation of the role of V1 INs in the regulation of flexor burst duration, and also found evidence that they regulate extensor activity in the absence of commissural input, possibly suggesting a more dynamic role for V1 INs in the generation of biomechanically advantageous flexion/extension asymmetry (Falgairolle and O’Donovan, 2019). Interestingly, the combined inactivation of both V1 IN and a subset of the V2 IN population (V2b) resulted in a total loss of flexor–extensor alternation (Zhang et al., 2014). Parallel examination of V2b INs determined that they inhibit extensor motoneurons in an analogous manner to the V1 INs with flexor motoneurons (Britz et al., 2015). These results appear to support the functional cooperativity of V1 and V2b INs to maintain the appropriate balance of flexor and extensor activity during locomotion.

Another ipsilaterally projecting population of propriospinal INs that has garnered interest in the context of locomotion is the Chx10-expressing V2a subpopulation. Unlike V1 and V2b INs, the V2a INs exclusively provide glutamatergic input in the mouse spinal cord (Al-Mosawie et al., 2007; Lundfald et al., 2007). The zebrafish homolog to the V2a population, identified by its expression of Chx10, is sufficient for generating locomotor rhythm (Ljunggren et al., 2014); however, this role is not entirely conserved in mammals. Optogenetic or synaptic blockade of the Shox2+ INs, some of which belong to the V2a population, perturbed locomotor rhythmogenesis in neonate mice (Dougherty et al., 2013), but ablation of only the Shox2+ V2a INs did not eliminate the mammalian spinal cord’s ability to generate rhythm. Surprisingly, V2a IN ablation created deficits in left–right alternation at intermediate to high speeds in adult mice (Crone et al., 2008, 2009), suggesting that the V2a INs, in fact, also innervate commissural INs responsible for left–right coordination.

While rhythmicity is conserved following midsagittal lesioning of the spinal cord, left–right coordination is lost, which is an important element of gait. Cross-talk between contralateral halves of the CPG is a critical feature of the locomotor network, where activity on one side can affect the contralateral locomotor output. This contralateral influence has been demonstrated through work involving split-belt treadmills (left and right limbs can be made to walk at different speeds), where compensatory alterations in phase duration were observed on the contralateral side when the speed was changed unilaterally (Frigon et al., 2013, 2017). Interestingly, this compensation occurs even in spinal cord transected cats, suggesting a propriospinal mechanism (Frigon et al., 2013, 2017). Indeed, the silencing of lumbar propriospinal neurons projecting from L2 to L5 in the adult rat was found to alter left–right coordination independent of speed (Pocratsky et al., 2017). These L2-to-L5 projecting propriospinal neurons consisted of both ipsilaterally projecting and commissural INs.

Commissural INs ensure proper coordination of both sides of the body during locomotion. These neurons may be contacted by V1 and V2a/V2b subtypes, potentially allowing them to coordinate shifts in flexion–extension across the left and right limbs during changes in speed. Commissural INs act by providing an excitatory or inhibitory drive to contralateral motor circuits. Their axons cross the midline at the same level as their cell bodies (Matsuyama et al., 2006) and then can project up and/or down the spinal cord (Eide et al., 1999; Stokke et al., 2002; Quinlan and Kiehn, 2007), even going as far as communicating between the cervical and lumbar segments (English et al., 1985; Matsuyama et al., 2004; Reed et al., 2006; Brockett et al., 2013; Ni et al., 2014; Mitchell et al., 2016; Ruder et al., 2016). One such population is the V0 INs, which are classified by their early expression of the developing brain homeobox 1 (DBX1). There are at least three subtypes of V0 INs: V0_D_ (delineated by paired box protein 7, PAX7), V0_V_ (express even-skipped homolog protein 1, EVX1), and V0_C_, which express paired-like homeobox transcription factor 2 (PITX2) and EVX1 (Moran-Rivard et al., 2001; Zagoraiou et al., 2009; Talpalar et al., 2013). Targeted ablation of DBX1-expressing neurons (all V0 INs) resulted in the loss of left–right alternation, giving way to synchronous hopping behavior at all frequencies of locomotion. Interestingly, selective loss of the V0_D_s has a more marked effect on coordination at slow locomotor speeds, whereas selective loss of the V0_V_s leads to loss of coordination at fast locomotor speeds (Talpalar et al., 2013). Unlike the other V0 populations that have clear roles in left–right coordination of locomotion, V0_c_s modulate motoneuron excitability through direct cholinergic inputs (Zagoraiou et al., 2009). The targeted ablation of the V0_C_s in adult mice did not have any measurable effect on the locomotor pattern but showed task-dependent modification of specific hindlimb muscle activity during swimming but not during walking (Zagoraiou et al., 2009).

The dI6 INs are another IN subtype involved with left–right coordination. They are marked by expression of either WT1, DMRT3, or Wt1/DMRT3, and form appositions with both ipsilateral and contralateral motoneuron pools (Andersson et al., 2012; Griener et al., 2017). A loss of functional DMRT3 led to an increase of dI6 INs exhibiting a Wt1+ identity and a drastic reduction in commissural projections, causing both the loss of coordination between the left and right CPGs, as well as a loss of coordination of the flexion–extension cycle in mice and horses. This evidence is consistent with the role of crossed inhibition in maintaining left–right alternation – as is the case for V0_D_ neurons – and also the role of ipsilateral inhibition in flexor–extensor balance (V1 and V2b INs).

One final ventral IN is the Sim1-expressing V3 population, a class of glutamatergic bilaterally projecting INs (Chopek et al., 2018). This population is highly heterogeneous in terms of their connectivity. V3 axons extend contralaterally, where they account for 22% of contacts on motoneurons positive for the excitatory neurotransmitter transporter VGluT2, and 27% of VGluT2 contacts on parvalbumin-positive Ia INs and Renshaw cells (Zhang et al., 2008). Silencing of V3 synaptic transmission using a cell-selective tetanus toxin light chain subunit (TeNT) expression system degraded locomotor pattern by increasing the variation in burst duration and interburst interval. Interestingly, the locomotor output was highly asymmetrical between the right and left flexor, increasing the burst duration of only one of two left–right L2 roots in the neonatal mouse spinal cord (Zhang et al., 2008). This latter finding may point to the involvement of V3 INs in stabilizing left–right alternation during walking. However, a subsequent study identified electrophysiological evidence of distinct V3 subpopulations in the spinal cord and suggested that differential recruitment of these subpopulations may occur during different modes of locomotion (Borowska et al., 2013). The dorsal V3 INs were primarily recruited during running episodes (in contrast to static and swimming conditions) when there is additional sensory feedback associated with greater mechanical loading. It was also found that these dorsal V3 INs received more inputs from sensory afferents than the ventral V3 population. Based on previous morphological examinations of commissural excitatory INs, the authors proposed that this dorsal V3 subpopulation may be responsible for relaying sensory information used to indirectly adjust left–right coordination, while the ventral V3 population presumably synchronizes motor outputs across multiple levels (Borowska et al., 2013). In a related fashion, computational modeling of the locomotor CPG identified V3 INs as a possible mediator of the transition from alternating to synchronous modes of locomotion with increasing speed (from trot to gallop to bound), but empirical evidence of this proposed function is currently lacking (Danner et al., 2017). A recent electrophysiological study uncovered a layered structure within the V3 subpopulation, where a ventromedial population may receive supraspinal locomotor commands while a ventrolateral population may relay these commands to different segments to coordinate several motor pools (Chopek et al., 2018). As such, V3 INs could reflect the dual roles of propriospinal INs to relay locomotor commands and to coordinate multiple components that form unit CPGs.

## Propriospinal Interneurons Coordinate Forelimb and Hindlimb Locomotor Networks

Separate CPGs regulate patterning of forelimb and hindlimb stepping, as evidenced by the ability of cervical and lumbar spinal cord segments to produce rhythmic oscillation independently of each other in the neonatal rat spinal cord (Ballion et al., 2001; Juvin et al., 2005; Gordon et al., 2008). However, the execution of smooth quadrupedal locomotion necessitates the coupling of forelimb and hindlimb motor outputs. This coupling occurs at the spinal cord level, as demonstrated by the coordinated rhythmic coupling of forelimb and hindlimb extensors during fictive locomotion in neonatal rodent isolated spinal cord preparations (Ballion et al., 2001; Juvin et al., 2005, 2012; Gordon et al., 2008). As such, the mechanism of this coupling is thought to occur either through inter-CPG communication via propriospinal INs or the integration of external sensory cues (Juvin et al., 2012). With regard to the former hypothesis, Juvin et al. (2005, 2012) found that the coupling of hindlimb and forelimb rhythm in neonatal rat fictive locomotor preparations was lost when a sucrose block was applied to the thoracic spinal cord, suggesting that direct connections between the CPGs are necessary to maintain coordination. Indeed, the existence of long propriospinal axons connecting these regions had long been hypothesized (Miller et al., 1973), and neuroanatomical evidence confirmed the presence of long ascending propriospinal connections from rostral lumbar spinal cord (Figure 1, long blue or upward red lines) to the cervical region along the ventrolateral funiculus (VLF) (Molenaar and Kuypers, 1978; English et al., 1985; Reed et al., 2006), with a majority of these neurons (85%) expressing VGluT2 (Brockett et al., 2013). The highest proportion of these ascending projections were ipsilateral (nine times more frequent than contralateral), and tended to be concentrated in the rostral lumbar segments (Brockett et al., 2013). Lesions to the thoracic VLF in the cat disrupt forelimb–hindlimb coupling (Brustein and Rossignol, 1998), further supporting the notion that these long ascending propriospinal INs maintain inter-CPG coordination. To test whether one CPG could govern the other via these propriospinal connections, Juvin and colleagues performed a midsagittal incision from C1 to T7 and found that left–right alternation was preserved when the connection to the caudal CPG remained, but subsequently disrupted when the caudal spinal cord was bathed in bicuculline and strychnine – GABA and glycine receptor antagonists – to disrupt the caudal locomotor pattern. In contrast, disruption of cervical left–right alternation through the addition of bicuculline and strychnine to the cervical spinal cord bath did not modify the lumbar pattern, suggesting the dominance of ascending propriospinal pathways in the regulation of forelimb/hindlimb coupling in the neonatal rat (Juvin et al., 2005).

While the long ascending propriospinal INs appear to play a major role in forelimb/hindlimb coordination, there is considerable evidence suggesting that long descending propriospinal INs (Figure 1, long green or downward red lines) also play a role in interlimb coordination (Matsushita et al., 1979; Skinner et al., 1980; Menétrey et al., 1985; Alstermark et al., 1987; Nathan et al., 1996). Ruder et al. (2016) used lumbar-injected retrograde *canine adenovirus-2* vectors in combination with a cervically injected adeno-associated virus (AAV) expressing the diphtheria toxin receptor, to selectively ablate lumbar-projecting cervical INs upon administration of the diphtheria toxin. Interestingly, they found that selective deletion of these long descending propriospinal INs resulted in the loss of coordination between hindlimbs, particularly at higher speeds (Ruder et al., 2016). More generally, the ablation of these neurons also reduced spontaneous locomotor speed and decreased the duration of locomotor bouts, which was accompanied or potentially compounded by the postural instability during locomotion that was observed. Examination of the neurotransmitter phenotype and developmental origin of long descending propriospinal INs revealed that those INs with connections to the highly rhythmogenic L2 region (Cazalets et al., 1995) are predominantly excitatory, with relatively minor representation of V2b INs (5.3%), and negligible V1 and V3 IN representation (Flynn et al., 2017). Similarly, Ruder et al. (2016) found a strong representation of excitatory V0 and V2 INs with very few V1, V3, and dI3 INs extending cervicolumbar projections. Even though the importance of forelimb/hindlimb coupling may be amplified in quadrupeds, there is circumstantial evidence that suggests this phenomenon also exists in bipedal animals. Coupling of bipedal arm and leg movements has been observed in upright walking (Dietz, 2002; Zehr et al., 2009, 2016; Frigon, 2017; Pearcey and Zehr, 2019), swimming (Wannier et al., 2001), and crawling (MacLellan et al., 2013) in humans, mirroring the expected behavior of paired oscillators observed in quadrupedal locomotion.

## Propriospinal Interneurons Integrate Sensory Information to Shape Locomotor Output

One of the critical features of mammalian locomotion is the integration of proprioceptive and cutaneous sensory information to guide locomotor output. Acute spinalized cats can spontaneously adjust to varying treadmill speed after administration of the noradrenergic agonist clonidine (Forssberg and Grillner, 1973). Other experiments using chronic spinal kittens found that spontaneous adjustment to speed could be performed even when individual limbs were subjected to different speeds using split-belt treadmills, a locomotor program analogous to turning (Forssberg et al., 1980; Frigon et al., 2013). These studies demonstrate the ability of the mammalian CPG to modify its locomotor pattern based on external sensory cues. While some of this can be explained by direct feedback from sensory afferents, integration of multimodal sensory feedback by propriospinal INs and rostrocaudal propagation of this signal may be required to generate complex sensory-induced motor responses involving multiple muscles (Levine et al., 2014). There are several specific phenomena that support this hypothesis. For example, the non-monosynaptic facilitation of motoneuron pools associated with stimulation of several lower limb nerves (common peroneal, posterior tibial, femoral) is thought to be mediated through short propriospinal INs situated rostral to the respective group of motoneurons (Chaix et al., 1997). Whereas long propriospinal INs could underlie the inter-limb modulation of reflexes such as the observations that static contralateral arm extension or flexion in humans produces soleus H-reflex facilitation or attenuation, respectively (Delwaide et al., 1977), while ipsilateral or contralateral sinusoidal arm movements depress soleus H-reflex excitability (Knikou, 2007).

While these findings strongly suggest a role for propriospinal INs in the integration and propagation of sensory feedback during movement, determining the identity and organization of the IN populations responsible is an ongoing endeavor. Several molecularly defined classes of spinal neurons described above have already been shown to receive sensory afferent inputs (e.g., V1, V3 INs). Neurons derived from dorsal progenitor domains are very likely to be involved in sensorimotor integration. For example, motor synergy encoder neurons, characterized by Tcfap2β and Satb1/2 expression, are involved in linking multiple motor pools together, and this muscle coordination seems to require sensory input (Levine et al., 2014). The dorsal IN type 4 (dI4 INs) mediate presynaptic inhibition of sensory terminals onto spinal neurons in order to properly gate sensory feedback to ensure smooth execution of movements in mice (Betley et al., 2009; Fink et al., 2014). Finally, the dorsal IN type 3 (dI3) INs seem another likely candidate for sensorimotor integration at the level of the spinal cord. The largely excitatory dI3 INs receive inputs from low-threshold cutaneous and proprioceptive afferents and extend projections to motoneuron pools within the cervical and lumbar enlargements (Bui et al., 2013, 2016). While no long descending propriospinal axons from cervical dI3 INs to lumbar segments have been found (Ruder et al., 2016), transsynaptic tracing experiments from the mouse quadriceps muscle found that dI3 INs project from adjacent lumbar segments (55% L1–L2, 6% L3, 39% L4–L6) to the flexor motoneuron pool, suggesting a moderately dispersed pattern of short propriospinal connectivity (Stepien et al., 2010). dI3 IN loss-of-function resulted in minor disturbances in locomotor gait in mice (Bui et al., 2016), which may speak to the distributed integration of sensory input among different spinal populations. More strikingly, their silencing attenuates recovery of treadmill stepping following SCI, implicating a specific population of spinal propriospinal INs in the recovery of motor function (Bui et al., 2016). As such, dI3 INs are one of the first populations of molecularly defined propriospinal INs that have been shown to be involved in the recovery of locomotor function following SCI. However, more generally, there is an abundance of literature implicating propriospinal INs in the recovery of locomotion after SCI, a topic that will be explored in depth below.

## Role of Propriospinal Neurons in the Recovery of Locomotor Function

Spinal cord injury can lead to a devastating loss of motor function. The severity of motor function loss depends on the location and nature of the injury, which dictates the degree of disruption of communication between the supraspinal centers controlling movement and spinal motor circuits. By virtue of their role in communicating higher motor commands to spinal circuits, a major focus of spinal cord repair has been the regeneration of descending tracts across the lesion site to restore lost motor input (Tuszynski and Steward, 2012). Accumulating evidence points to propriospinal INs as an additional target for promoting the recovery of locomotor function (Taccola et al., 2018; Loy and Bareyre, 2019). As a consequence of their position within the spinal cord and their central role in the generation of locomotion, propriospinal INs are well-situated to propagate supraspinal commands to motor systems below the level of injury. Furthermore, the shorter distance required for propriospinal axons to bridge the lesion compared to axons from cortical or brainstem neurons make them a more straightforward target for regenerative approaches. Several changes to neural circuits involving propriospinal INs have been observed that could facilitate the recovery of locomotor function.

### Propriospinal INs Receive New Connections From Supraspinal Neurons

New synaptic contacts made by descending tracts onto propriospinal INs following SCI have been repeatedly demonstrated in animal models of SCI. Seminal work by Bareyre et al. (2004) revealed that spontaneous sprouting of axon collaterals from the corticospinal tract (CST), in particular, those contacting propriospinal INs, could promote recovery of locomotion after SCI. After a mid-thoracic dorsal transection of CST axons in rats, lesioned CST axons spontaneously sprouted collaterals into the cervical gray matter where they made new connections with both short and long propriospinal INs. Remarkably, the CST projections onto short propriospinal INs, which did not bridge the lesion site, were lost 12 weeks after injury while the CST contacts made with the long propriospinal INs crossing the lesion site were maintained. In addition, the number of direct contacts between long propriospinal axon terminals and lumbosacral motoneurons was doubled 8 weeks after the dorsal hemisection. The maintenance of these intraspinal pathways from cervical to lumbar segments following the dorsal hemisection was verified by pseudorabies virus tracing and their functionality was supported by EMG signals in the hindlimb evoked by intracortical microstimulation. Similarly, lesioning of the reticulospinal tract (RtST) by a unilateral cervical hemisection in adult rats was shown to increase reticulo-propriospinal contacts from damaged RtST axons (Filli et al., 2014). Since brainstem locomotor pathways such as the RtST are critical in control of locomotion in mammals (Brownstone and Chopek, 2018), the remodeling of reticulospinal pathways involving propriospinal INs may be an important factor in locomotor recovery (Asboth et al., 2018).

### Propriospinal INs Form Detours Around Lesions

One reason why descending inputs may be increasing their connections with propriospinal INs after an injury is the ability of these spinal neurons to form circuits, which may be pre-existing or *de novo*, that circumnavigate spinal lesions to provide an alternative flow of motor commands to spared lumbar circuits for locomotion. This hypothesis has been tested, in particular, using the staggered hemisection injury paradigm (Kato et al., 1984; Courtine et al., 2008; May et al., 2017). In these studies, an initial hemisection is made to sever ipsilateral descending pathways on one side of the body (Figure 2B). A second hemisection is subsequently made on the other side of the body at a more rostral thoracic segment to disrupt spared descending pathways contralateral to the first hemisection. In some studies, the second hemisection is made immediately or after a delay, the latter to determine whether the earlier hemisection was followed by adaptations to spinal circuits that may lead to spontaneous recovery of stepping function (Courtine et al., 2008; May et al., 2017). When an initial hemisection at T12 was followed 10 weeks later by a second contralateral hemisection at T7, greater recovery of locomotor activity was observed compared to animals that received the contralateral hemisections at T7 and T12 simultaneously (Courtine et al., 2008). The improvements in recovery when hemisections were made with a delay as opposed to simultaneously suggests that the initial hemisection promoted remodeling of propriospinal circuits to form detours around this initial hemisection that were not disrupted by the second, delayed, contralateral hemisection (Figure 2C). Locomotor recovery following the delayed staggered hemisections was associated with evidence of connectivity between spared lumbar circuits with propriospinal INs in the intervening region between the lesions (T8–T10) while showing virtually no direct connectivity between supraspinal locomotor nuclei and lumbar circuits. Furthermore, an excitotoxic ablation of T8–T10 neurons eliminated any observed spontaneous recovery, supporting the idea that propriospinal INs had formed detours to permit the transmission of descending information to hindlimb CPGs (Courtine et al., 2008).

The possibility of detour circuits formed by propriospinal INs was further strengthened by evidence of increased connectivity between descending RtST tracts and propriospinal INs following a staggered contralateral hemisection protocol where one hemisection at T10 eliminated the ipsilateral RtST and CST followed by an over-hemisection at T7 removing the contralateral RtST and both CSTs (May et al., 2017). The number of RtST contacts onto propriospinal INs was significantly higher if both hemisections were made with a delay, suggesting again that locomotor commands were routed through detour circuits formed by propriospinal INs after the first hemisection.

### Propriospinal INs Relay Sensory Feedback to Activate Spinal Locomotor Circuits

The formation of detours around spinal lesions is not possible after complete SCI, and so far, axonal regeneration across lesion sites remains a significant challenge. However, animal models of complete SCI often exhibit some recovery of locomotor function despite a lack of any meaningful regeneration across lesion sites. This recovery is believed to involve plasticity in spinal circuits below the lesion site (Barrière et al., 2008; Rossignol and Frigon, 2011; Martinez et al., 2012) and propriospinal INs are poised to play significant roles in promoting this recovery due to their ability to communicate across spinal segments.

Activation of sensory feedback in the days following complete SCI has repeatedly been shown to be crucial to this recovery of locomotor function (Bouyer and Rossignol, 2003; Lavrov et al., 2008; Sławińska et al., 2012; Takeoka et al., 2014). Propriospinal INs that integrate sensory feedback may be particularly central to promoting locomotor recovery by relaying sensory feedback to the spared CPGs distributed across the lumbar spinal cord. For example, dI3 INs, which integrate cutaneous and proprioceptive inputs, have been shown to be involved in the recovery of locomotor function following complete SCI. dI3 IN loss-of-function significantly depressed the ability to generate sensory stimulus-induced locomotor activity in a reduced spinal cord locomotor preparation and largely eliminated the benefits of treadmill training after SCI (Bui et al., 2016).

With increasing evidence that propriospinal INs may play an invaluable role in promoting recovery of locomotor function, several different approaches have been explored that center on these neurons. We describe these strategies below.

## Therapeutic Strategies for SCI Utilizing Propriospinal INs

### Stimulation of Propriospinal INs to Augment Activity After SCI

Although many neurons and cells die after SCI around the lesion site, some neurons survive the injury but may become dormant, leading to a state where voluntary movements are absent. These neurons may still have the capacity to become excited in response to convergent inputs from spared descending tracts and sensory feedback, but so far, in human SCI patients with sustained loss of movements, this has only been observed when combined with electrical neuromodulation and/or pharmacological interventions (Gerasimenko et al., 2007; Courtine et al., 2009; Harkema et al., 2011; Angeli et al., 2014). Pharmacological strategies aimed at enhancing propriospinal IN activity promote locomotion by facilitating the transmission of motor commands through propriospinal relays, and by increasing the excitability of downstream locomotor networks.

This particular approach stems from experiments using *ex vivo* spinal cord and brainstem–spinal cord preparations, wherein fictive locomotor activity could be induced using a variety of physiological and pharmacological manipulations to achieve a state of increased excitability in the locomotor network. Transient elevations in extracellular potassium or application of neurotransmitter agonists restored locomotor activity elicited by brainstem stimulation following a staggered hemisection paradigm to sever all direct bulbospinal connections in a neonatal brainstem–spinal cord preparation (Zaporozhets et al., 2011), suggesting that dormant propriospinal relays could be activated with the appropriate stimulus. Subsequent testing of neurotransmitter agonists, including serotonin, dopamine, and norepinephrine, successfully induced locomotor activity when applied to thoracic segments in spinal cord preparations with staggered contralateral hemisections at T1/T2 and T9/T10. Glutamatergic agonist NMDA only promoted locomotor activity in this experimental paradigm when applied with serotonin, while acetylcholine did not promote locomotor activity. *In vivo* animal studies have also demonstrated the efficacy of similar pharmacological approaches in promoting locomotor activity following SCI. This was first demonstrated in acutely spinalized cats injected with L-DOPA, where it was observed that stimulation of flexor reflex afferents could elicit ipsilateral flexor and contralateral extensor activity reminiscent of normal locomotor patterning (Jankowska et al., 1967). More recent iterations of this approach have focused on serotonin receptor agonists such as quipazine and 8-OH-DPAT, typically delivered in combination with step training and/or epidural stimulation to promote locomotor recovery in rodent models of SCI (Fong et al., 2005; Gerasimenko et al., 2007; Ichiyama et al., 2008; Courtine et al., 2009; Cowley et al., 2015; Duru et al., 2015). Epidural stimulation, which is often applied through the use of microelectrode arrays implanted over the lumbosacral cord (Lavrov et al., 2008; Angeli et al., 2018; Capogrosso et al., 2018; Wagner et al., 2018), does not explicitly target propriospinal INs. However, the ability of epidural stimulation to facilitate the production of hindlimb stepping after SCI seems to be a consequence of the recruitment of propriospinal INs along with MNs by the activation of sensory afferents (Capogrosso et al., 2013; Moraud et al., 2016; Formento et al., 2018). Therefore, while these mixed chemical and electrical interventions are not entirely specific to propriospinal INs and are likely to have broader effects on locomotor network excitability, it should be noted that significant locomotor recovery was observed when quipazine was locally injected to target propriospinal relays in staggered hemisection models at T2–T4 and T9–T11 segments (Cowley et al., 2015).

Propriospinal INs in lumbar segments, well below the injury site, may also be targeted for stimulation. For example, silencing dI3 INs has been shown to shunt recovery of stepping following a complete transection (Bui et al., 2016). It follows that stimulating spared dI3 INs in the lumbar spinal cord may be effective in reviving CPG activity in the absence of descending locomotor commands. Stimulation of intact propriospinal INs linked with vertebrate CPG rhythmogenesis such as V2a INs (Crone et al., 2009; Ljunggren et al., 2014), and Shox2+ INs [non-V2a subpopulation (Dougherty et al., 2013)] could also be useful for activating CPGs in the injured spinal cord. Direct stimulation of these populations of propriospinal INs or enhancing their plasticity to promote connectivity with detour circuits could be part of effective therapeutic approaches to restore lost locomotor function.

While these general stimulation approaches hold great promise or have already shown some efficacy in restoring function, a better understanding of the relevant propriospinal IN populations may result in more effective treatment strategies. For example, specific optogenetic stimulation of V3 INs in a mouse model of sacral SCI produces muscle spasms putatively generated by a disinhibited or hyperexcitable locomotor network (Lin et al., 2019). Inhibiting V3 INs was found to reduce the appearance of these muscle spasms; however, subthreshold stimulation of these same neurons also prevented sustained muscle spasms, but would theoretically enable them to be recruited for locomotor functions (Lin et al., 2019). The varied effects of V3 IN manipulation, from detrimental to beneficial, emphasize the need for a better understanding of the contributions of different populations of propriospinal INs to locomotor recovery after SCI.

### Disinhibition of Propriospinal IN Activity Following SCI

Enhancing the activity of propriospinal INs, in particular, those involved in bypassing or circumventing a lesion appears to be a promising strategy. However, a recent study suggests that downregulating the excitability of spinal inhibitory INs may enable increased activity in propriospinal circuits, which ultimately leads to improved recovery of locomotor function (Chen et al., 2018). Systemic delivery of CLP290, an agonist of the neuron-specific K^+^/Cl^–^ co-transporter KCC2, improved locomotor recovery in a staggered hemisection paradigm in adult mice. Interestingly, selective expression of KCC2 in GABAergic, but not glutamatergic or cholinergic neurons of the spinal cord, resulted in the sustained recovery of hindlimb stepping. Expressing KCC2 in GABAergic neurons seemed to increase the activity of propriospinal INs between the staggered hemisections, suggesting that generalized increases in activity of spinal circuits involving propriospinal INs may not be effective without concomitantly decreasing the influence of inhibitory neurons gating the flow of descending commands through propriospinal relays.

More generally, locomotor network disinhibition has also been shown to promote locomotor activity following spinal cord transection in cats. Using this experimental model, it was found that untrained cats or those with poor stepping function could be prompted to execute successful stepping after the administration of either glycinergic receptor antagonist strychnine, or GABA_A_ receptor antagonist bicuculline (Robinson and Goldberger, 1986; Edgerton et al., 1997; de Leon et al., 1999). Interestingly, these strategies to block inhibitory synapses did not yield significant benefit in animals that had previously received step training, suggesting that either a plateau locomotor function had already been reached, or that the step training itself influenced the function of inhibitory synapses in the locomotor network.

### Promoting Propriospinal Plasticity Following SCI Through Physical Activity

Physical rehabilitation with treadmill-aid or robotic assistance has been shown to increase recovery of hindlimb stepping beyond the levels of spontaneous recovery (Dietz et al., 1995; Goldshmit et al., 2008; Edgerton and Roy, 2009; Harkema et al., 2011, 2012; Rossignol and Frigon, 2011; Martinez et al., 2012; Angeli et al., 2014; Gill et al., 2018; Wagner et al., 2018). Animal models of SCI provide evidence that this activity-dependent recovery could be associated with changes in connectivity to propriospinal circuits or reorganization of propriospinal circuitry (Theisen et al., 2017). Anatomical tracing experiments (van den Brand et al., 2012) provide evidence that overground training in rats following staggered hemisections at T7 and T10 increases CST projections onto T8/T9 spinal INs that are putative propriospinal INs. Locomotor training has also been shown to partially reverse the loss of cholinergic innervation of motoneurons, putatively from propriospinal cholinergic INs (the V0_C_ INs, Zagoraiou et al., 2009), following SCI (Skup et al., 2012).

Sensorimotor integration involving propriospinal INs may be modified by step training (Cote, 2004; Knikou and Mummidisetty, 2014). Certainly, proprioceptive feedback activated during locomotor training seems to be essential for the establishment of detour circuits and locomotor recovery (Takeoka et al., 2014). Hindlimb proprioception, in particular, was found to be critical for the guidance of propriospinal detour circuits following a thoracic hemisection, as hindlimb-targeted ablation of proprioceptive afferents yielded a similarly reduced number of propriospinal connections compared to a more generalized ablation of proprioceptive afferents (Takeoka and Arber, 2019). Interestingly, forelimb activity has been linked to the recovery of hindlimb locomotor control in incomplete SCI (Shah et al., 2013). Training of both forelimbs and hindlimbs of rats with a thoracic hemisection had a greater impact on recovery than training hindlimbs alone (Shah et al., 2013). Quadrupedal step training activated sensory inputs from the forelimbs, which in turn seemed to increase the number of thoracic propriospinal INs rostral to the lesion site that projected to upper lumbar segments. These results and others suggest a complexity in the influence of forelimb and hindlimb sensory activation in promoting plasticity of both descending and ascending propriospinal circuits following SCI (Côté et al., 2012).

While physical rehabilitation seems to promote recovery of hindlimb stepping, levels of recovery have yet to be optimized (Torres-Espín et al., 2018). A number of approaches have been attempted to increase recovery levels further. Recent studies have reported that promoting recovery through electrical and pharmacological stimulation can augment the extent of recovery derived from locomotor training in rodent models of SCI (Asboth et al., 2018). Indeed, the combination of locomotor step training, epidural stimulation, and injection of dopaminergic and serotonergic agonists increased recovery of locomotor activity, including volitional locomotion, after the loss of CST innervation resulting from spinal contusion injury. Neuroanatomical tracing, in combination with optogenetic and/or chemogenetic manipulations, suggests that this recovery results from cortico–reticulo–spinal reorganization that includes increased synaptic contact from the motor cortex to the ventral gigantocellular reticular nuclei, and from the ventral gigantocellular nuclei to putative propriospinal INs (Asboth et al., 2018).

### Promoting Axonal Growth Across the Lesion

Propriospinal INs exhibit the capacity to regenerate their axons across lesion sites in the spinal cord. Commissural INs, some of which are likely to be propriospinal, have been shown to form crossing connections across the lesion site following midsagittal spinal sections through their axons (Fenrich et al., 2007; Fenrich and Rose, 2009). Immunostaining and electrophysiology were used to demonstrate that newly formed synapses from these commissural INs were functional and could evoke potentials in contralateral motoneurons. Therefore, at least some propriospinal INs would seem to have the capacity to grow through the inhibitory environment of spinal lesions.

Promoting regrowth of propriospinal INs may be facilitated by overexpression of growth-associated genes or application of neurotrophic factors such as brain-derived neurotrophic factor (BDNF) and NT-3, which have been shown to promote sprouting and rewiring of neurons at the injury site (Bradbury et al., 1999; Hammond et al., 1999). Application of BDNF to the left motor cortex after a thoracic over-hemisection in mice caused collateral sprouting of injured CST axons and formation of contacts with propriospinal INs that was accompanied by functional recovery (Vavrek et al., 2006). In another study, the transduction of the transcription factor Kruppel-like factor 7 (KLF7) by an AAV vector above the injury site after a T10 contusion was shown to promote both descending propriospinal axon plasticity and synapse formation after a T10 contusion in adult mice (Li et al., 2017). KLF7 regulates the expression of a number of genes, including the neurotrophin NGF and its receptor TrkA (Caiazzo et al., 2010). This enhancement of propriospinal plasticity by KLF7 overexpression was associated with significantly improved motor function.

The regenerative capacity of axons of injured propriospinal INs and descending motor axons can be further enhanced by substrates that promote regrowth. For example, peripheral nerve grafts, in combination with the neurotrophin glial cell line-derived neurotrophic factor (GDNF) and the enzyme chondroitinase ABC to degrade inhibitory chondroitin sulfate proteoglycans within lesion scars, promoted the extension of spinal axon processes within and across the graft (Tom et al., 2009). A more recent study suggests that the application of specific growth factors selected to increase the intrinsic capacity of propriospinal INs for axonal growth, to induce the expression of growth-supporting substrate, and to provide guidance cues for regenerating axons, could be sufficient to induce axonal regeneration across a lesion (Anderson et al., 2018). Delivery of osteopontin, insulin-like growth factor 1, ciliary-derived neurotrophic factor, fibroblast growth factor 2, epidermal growth factor, and glial-derived neurotrophic factor through a combination of AAV injections or synthetic hydrogels near and in the lesion site promoted the growth of propriospinal INs through a spinal lesion in both a rat and mouse complete SCI model. While behavioral improvements observed using this strategy were relatively minor, the new propriospinal projections appeared to be capable of conducting action potentials across the lesion site, opening the door to future strategies to improve the integration of these axons into motor circuitry. While these strategies hold promise, the heterogeneity of propriospinal INs (i.e., short versus long-projection), as well as the type and location of the spinal cord lesion are likely to influence the gene-expression and substrate requirements for successful regeneration (Conta and Stelzner, 2004; Swieck et al., 2019).

The identification and application of multiple molecules to coax the regrowth of descending and propriospinal axons across the lesion site and toward spinal locomotor circuits caudal to the lesion site have led to promising results. However, there is still a significant gap between the level of recovered motor function in treated SCI animals and locomotor function in the intact state. A recent study highlights the possibility of promoting propriospinal axon plasticity through the engagement of the components of the motor circuit downstream from descending inputs and propriospinal INs, namely motoneurons and the musculoskeletal system. Motoneurons have extensive dendritic arborizations, some of which even extend beyond the gray matter into the white matter (Rose and Richmond, 1981; Keirstead and Rose, 1983; Tosolini and Morris, 2016). This broad coverage of the ventral spinal cord could permit motoneurons to have far-ranging influences on regenerating processes after SCI, perhaps through the release of BDNF (Ying et al., 2005; Joseph et al., 2012). While motoneuron dendrites undergo atrophy after SCI (Gazula et al., 2004), the delivery of AAV expressing NT-3 to locomotion-associated motoneurons via transiently demyelinated sciatic nerves after a T10 contusion in mice reduced motoneuron dendrite atrophy. This reduced atrophy was accompanied by an increased presence of descending inputs from spared propriospinal INs onto MNs within the L2–L5 spinal cord levels when compared to non-treated controls and resulted in modest improvements in locomotor behavior (Wang et al., 2018). Interestingly, motoneuron dendrite atrophy after SCI is reduced by exercise (Gazula et al., 2004), further suggesting that combining the application of molecules to increase regenerative properties of propriospinal INs, whether directly or through the influence of other components of the locomotor network, with physical activity could produce greater levels of functional recovery.

Finally, several studies suggest that the benefits of promoting axonal growth of propriospinal axons through growth-stimulating molecules can have unintended consequences. These secondary complications have been illustrated, in particular, for BDNF. AAV delivery of BDNF in rats spinalized at thoracic level improved weight support and treadmill walking over untreated SCI animals (Ziemlińska et al., 2014). However, increased excitability of spinal locomotor circuits eventually resulted in increased clonic movements in BDNF-treated animals over time. This was potentially due to the upregulation of glutamatergic (vGluT2) and GABAergic (GABA, GAD1, and GAD2) transmission in combination with reduced levels of the potassium chloride co-transporter, KCC2, which alters the reversal potential of chloride channels associated with GABA/glycinergic synapses causing them to become depolarizing instead of hyperpolarizing. Similarly, another study reported that BDNF overexpression through viral delivery and/or cell grafts in a cervical hemisection rat model led to spasticity-like symptoms such as clenching of the paws and sustained wrist flexion (Fouad et al., 2013). Motor circuits of the spinal cord are not the only ones that can be modified by BDNF, as viral delivery of BDNF following complete thoracic transection in adult rats promoted stepping function but also increased pain sensitization (Boyce et al., 2012). Therefore, these results emphasize the need for tight regulation of the timing of delivery and the dosage of growth-promoting molecules in order to avoid secondary complications.

### Strategies Aiming to Generate New or Replace Lost Propriospinal INs

An alternative approach to modulating the activity of existing propriospinal INs or promoting remodeling of propriospinal circuits would be to replace lost propriospinal INs due to injury (Bonner and Steward, 2015; Courtine and Sofroniew, 2019). Recently, neural stem cell transplantation has gained much attention as a repair mechanism after SCI. Transplantation of neural stem/progenitor cells (NSPC) was shown to promote functional recovery after mild and moderate contusion at the T9 level in mice by reorganizing the circuitry of propriospinal INs (Yokota et al., 2015). Retrograde transsynaptic tracing showed that propriospinal circuits reorganize in both longitudinal and transverse directions, enhancing synaptic integration between the engrafted NSPCs and the host neurons. Recently, human neural stem cell grafts into C7 hemisection lesion sites in non-human primates were shown to extend axons into the caudal spinal cord of the host. These same grafts exhibited evidence of integration with descending tracts from supraspinal motor centers, a feature that was associated with improvements in forelimb function (Rosenzweig et al., 2018). As such, these stem cell interventions provide a promising strategy to generate new propriospinal relays in cases where spared motor circuitry is insufficient to restore meaningful motor function after SCI.

## Perspectives

As demonstrated by the research described herein, propriospinal INs are necessary for the appropriate control of locomotion in the intact state. It is therefore unsurprising that propriospinal INs are also critical to locomotor recovery after SCI, and represent important therapeutic targets for novel SCI treatments. However, given the diversity of propriospinal INs in organization and function as well as the neural dysfunction that can arise from neural plasticity following SCI (Boyce et al., 2012; Beauparlant et al., 2013; Fouad et al., 2013; Ziemlińska et al., 2014), effectively harnessing propriospinal INs will likely require significant advancement in our understanding of how these neurons adapt to SCI, and how different populations work together to support locomotion after a substantial loss of supraspinal input. New methods to non-invasively manipulate propriospinal INs, either regionally using epidural stimulation, or in a population-specific manner using chemogenetic and optogenetic approaches (Asboth et al., 2018; Mondello et al., 2018; Lin et al., 2019), will be critical to develop new therapeutic strategies and to improve our understanding of propriospinal IN function after SCI. Supplemental strategies to improve integration of propriospinal IN relays, such as the transcranial stimulation approach proposed to induce greater CST plasticity to propriospinal INs (Krishnan et al., 2019), or more conventionally, locomotor training paradigms to refine these propriospinal circuits, may also prove to be critical for the optimization of locomotor recovery. In any case, as the knowledge of propriospinal INs and locomotion steadily advances, the future of the field appears bright – with significant challenges and opportunities for discovery on the horizon.

## Author Contributions

AL, SG, NL, and TB wrote the sections of the manuscript, contributed to the manuscript revision, and read and approved the submitted version. NL and TB designed the figures.

## Conflict of Interest

The authors declare that the research was conducted in the absence of any commercial or financial relationships that could be construed as a potential conflict of interest.
